# Experiments and Observations on the Putrefaction of Flesh in Different Kinds of Gas

**Published:** 1803-03-01

**Authors:** C. H. W. Bockmann

**Affiliations:** Carlsruhe


### EXPERIMENTS *and* OBSERVATIONS *on the* PUTREFACTION *of* FLESH *in different Kinds of* GAS; *by Professor* C. H. W. BOCKMANN, *of Carlsruhe.*

THE phenomena of putrefaction, which are observed on animal substances in general, but particularly when exposed to different species of gas, have engaged the attention of many naturalists and chemists, and a number of experiments have been made, for the purpose of ascertaining the several facts attending so remarkable a process. Among the chemists who have chiefly devoted their researches to this subject, Dr. Priestley and Signor Brugnatelli deserve to be ranked first, having made a series of experiments on the putrefaction of animal substances in atmospherical air, carbonic gas, hydrogen gas, and nitrous gas. The former gentleman found, that on exposing a dead mouse to putrefaction in atmospherical air, the mass of air was at first increased, but diminished afterwards by the fourth or fifth part, whereby some carbonic gas was disengaged. Farther, by the putrefaction of mutton and beef, hydrogen gas was evolved; and a mouse, placed in carbonic gas, passed over into a state of putrefaction about a month after. Putrid mutton yielded likewise hydrogen gas in this gas. Above all, the nitrous gas was found to prove particularly antiseptic; as mice, preserved in it during the whole month of July, had neither received a putrid smell nor showed any other symptoms of putrefaction, their flesh being almost fresh, and quite solid; whereas the flesh of a pigeon began to change its smell and taste after being suffered to remain in it for several months; and though these could not properly be called called putrid, and the flesh had still remained of a solid consistency, yet when roasted it could not be eaten; pigeons, however, preserved in nitrous gas for longer than six months, emitted a fetid stench, the flesh remaining solid; and, lastly, Mr. Priestly observed, that hydrogen gas appeared not to stop the progress of putrefaction. Signor Brugnatelli proved, however, by his experiments, that flesh could be preserved in hydrogen gas for several days, and even for some months, without being rotten; on which account he considers this gas as antiseptic.

Notwithstanding these facts and observations, I have thought it not improper to repeat those experiments of Priestley and Brugnatelli, to continue them in a different manner, and to ascertain more precisely the phenomena which flesh exhibits in single gasses, and in their different mixtures, during the several periods of putrefaction; for which purpose I have made the following experiments:

Exp. 1. Bottles of white glass, holding about 8 or 10 cubic inches of fluid, were filled with different species of gas; of these, the bottle signed A, contained *atmospherical air;* B, *oxygen gas,* prepared from manganese; C, *azotic gas,* separated from atmospherical air; D, *hydrogen gas,* prepared from diluted sulphuric acid and iron filings; E, *carbonic gas,* prepared by chalk and diluted sulphuric acid; F, *nitrous gas,* prepared by diluted nitrous acid and copper wire; G, half of *nitrous gas* and half of *carbonic gas.* The openings of these bottles were shut under water with corks, on which were fastened pegs of wood with small pieces of beef. Some minutes after the flesh in B, F, and G, grew evidently redder. The bottles were put into a vessel with water, which being covered with a plate of white iron, was exposed to the action of the sun in the first days of July. The following morning the flesh in F and G was of a beautiful red; towards the evening it appeared in B, white, yellowish, and somewhat putrid; in C, D, and E, however, but particularly in F and G, it remained red. On the morning of the third day, I found no considerable alteration; but towards the evening of the same day, the flesh in B appeared putrid, and also in A it had acquired a bluish putrid colour. On the evening of the fourth day, the flesh in F was of the most vivid red colour; next to it came that in G; then the flesh in E. In D and C it had the same colour, which was less vivid than in the other bottles. In A, the flesh was of a bluish red, and a little brownish; in B, it was putrefying, of a yellow, brown, and bluish colour; some water had entered ered this bottle. On the sixth day, I found the flesh, particularly in F and G, and also in C, E, and D, to be still red; in A, and principally in B, it was very putrid; more water had entered this bottle. On the eighth day, the putrefaction in A and B was considerably increased; the flesh was entirely surrounded with water. In the rest the colour appeared a little darker, which, however, at least, in F and G, seemed not to depend on a beginning putrefaction, but it appeared to be dried by the heat of the sun; in C, D, and E, the colour was rather bluish red. In A, the colour of the flesh was blue, and it looked putrid. The flesh in B, being no more in contact with the gas, I took it out; it had a very strong putrid stench, and a white colour resembling animal fat. The water was of a yellow greenish colour, and reddened the blue vegetable colour, but the redness disappeared by degrees. The gas was diminished to a sixteenth part of its former volume, &c. On the ninth day, the putrefaction had increased in A, and the flesh in the other bottles became darker. After ten days the flesh in C, D, E and F had grown darker, and was almost dried up; in G it had retained a red colour, but in A it was moist, dark blue, and of a putrid appearance. It was not altered considerably afterwards, except that it was somewhat dried. Several weeks after the pieces of flesh were taken out of the bottles, they had no remarkable smell, but when moistened with water they emitted more or less of a peculiar caseous smell. The heat which had acted on the flesh during this experiment was between 10 and 14° Reaum. The middle temperature was about 14—16°. Several times I had also exposed the vessels to the full action of the sun.

Exp. 2. I filled several small bottles, each holding 12 to 15 cubic inches, with the following kinds of gas: 1. Nitrous gas. 2. Hydrogen gas. 3. One half of nitrous gas and half of carbonic gas. 4. Carbonic gas. 5. Atmospherical air. 6. Oxygen gas. 7. Azotic gas. Having brought into them pieces of flesh, in the same manner as had been done in the former experiments, I observed a remarkable redness in the flesh which was placed in nitrous gas, oxygen gas, and also, in some measure, in the carbonic gas. On the second day, the flesh in 1, 3, 4, and 7 was of a vivid red colour, and likewise in 5. In 2 it looked greyish black; and in 6 whitish yellow. On the third day, the flesh in 6 had already a putrid appearance; in 5 it had only grown a little bluish, but was not yet putrid. On the fourth day, it was grey yellowish in 6, and very putrid.

In

In 5 it began to grow putrid, whereas in 1 it was still beautifully red; in 2, 3, 4, and 7 it was less red than in 1. On the sixth day I found the flesh in 1, 2, 3, 4, and 7 to be no farther altered; in 5 however it looked brown and putrid; in 6 it was quite rotten. On the eighth day, it was in 1, 2, 3, 4, and 7, of a bluish red colour, somewhat dry, but without putrefaction. In 5, and particularly in 6, it had a dark grey colour, and was almost liquid. On the following days, the remainder of the flesh in 5 and 6 was of a blackish colour; the rest was dark red, and dried up. On being taken out of the vessels several weeks after, the pieces appeared as in the former experiment; both experiments were made at the same time.

Exp. 3. The bottles being again filled with different mixtures of gasses, pieces of beef were introduced in the same manner as before; namely, there was contained in A hydrogen gas; B oxygen gas; C equal parts of hydrogen gas and of carbonic gas; D equal parts of azotic gas and of carbonic gas; E equal parts of hepatic hydrogen gas and of carbonic gas; F equal parts of oxygen gas and of carbonic gas; G equal parts of nitrous gas and of hydrogen gas; H equal parts of hepatic hydrogen gas and of azotic gas. The pieces of beef in B and G grew soon considerably redder; in E and H however they received a yellowish colour, while in the other bottles it experienced no considerable alteration. Second day, the flesh contained in A C and G had of all the reddest colour; it was less red in F and B; in H and E its colour was yellowish and greyish brown. Third day, the flesh in A C and G was still of a beautiful red; somewhat less in D; whereas in B it looked putrid and brownish; in the other vessels its appearance was not visibly changed. Fourth day, in G it was yet fresh and of a bloody colour; it appeared less red in A and C; in E and H the colour was grey; in B blackish and marks of putrefaction were evident; in F it looked dry and dark brown. Fifth day, the flesh appeared to be dry on the whole; it had the finest red colour in G, whereas in A C and D the colour was rather more or less dark red; in E and H it was grey, blackish; in B and F dark brown. Eighth day, the red colour of the flesh in A C D and G had grown darker, but finding no change to have taken place in the pieces of flesh contained in the other bottles, I determined to examine the remaining pieces. The flesh in A showed no marks of considerable putrefaction, it being still red but dry, and therefore of a dark colour; no considerable smell; which however was likewise to be perceived in the the remaining gas. On applying a candle to the gas, it took fire without any explosion; phosphorus did not fume nor shine in it. The flesh taken from the bottle B had a yellowish black colour, a putrid stench, and its volume had considerably decreased. The remaining gas made lime-water turbid, a candle burned in it, &c. The flesh in the bottle C looked fresh, had but a slight, not quite putrid smell; by being shaken with lime-water it diminished about one half of its volume; phosphorus shone in the remaining gas, and the gas took fire without any explosion. The smell of the flesh in D was inconsiderable; the remaining gas troubled the lime-water, and extinguished a burning candle. The surface of the flesh that had been contained in E was greyish black, smelling like hepar sulphuris, but no other marks of putrefaction were perceived; the gas was absorbed by lime-water, and extinguished a candle. The flesh in F was of a yellowish brown colour, solid, but its smell was putrid; lime-water was troubled by the remaining gas. The flesh in the vessel G was solid, red, and without any putrid smell; lime-water became only a little turbid by the gas, and it was inflamed without explosion. In H the flesh was greyish, solid, and without any remarkable smell; the gas emitted an hepatic smell, and took no fire. During this experiment the temperature was between 10° and 30° Reaum.; the mean temperature amounted to $14\frac{1}{2}$° Reaum.

Exp. 4. The bottles being again filled with different gases, contained, No. 1. Hydrogen gas. 2. Two-thirds hydrogen gas and one-third oxygen gas. 3. Half hydrogen gas and half oxygen gas. 4. One-third hydrogen gas and two-thirds oxygen gas. 5. One-sixth hydrogen gas and five-sixths oxygen gas. 6. One-ninth hydrogen gas and eight-ninths oxygen gas. 7. Oxygen gas. The pieces of beef that were introduced into the vessels were quite fresh, and some time after I observed the flesh in 1 to have received a beautiful red colour, decreasing in the other vessels in proportion as to the less oxygen that they contained. Second day, I found the flesh in 1 still red, but the colour had become less red in the other vessels, particularly in 7; the pieces in 2, 3, 4, and 5, were the same, but 6 approached more to 7, which had a yellow putrid appearance. Third day, in the evening, I found the flesh in 1 to be still fresh and red; in 2 it was darker; but in 3, 4, and 5 it appeared putrid; in 7 the putrefaction seemed to be particularly great, but on the whole there was no great difference in the last six bottles with regard to the consistency and and colour of the flesh contained in them. Fourth day, the flesh in 1 was not particularly changed, but that in the other vessels was red, brown, and putrid. The temperature had been between 12 and 30° Reaum. Six weeks after, I found the flesh to have more moisture than it had on the fifth day; in 1 it was in some places light red, in others yellowish brown; in the other vessels it was yellow, brown, blackish and very putrid. I preserved the flesh in these bottles for eighteen months, without observing any visible alteration.

Exp. 5. About the time I was making the fourth experiment I filled some other glass bottles, each containing 14 cubic inches, with the following kinds of gas. No. 1. Half nitrous gas and half carbonic gas. 2. Half azotic gas and half carbonic gas. 3. Half hydrogen gas and half carbonic gas. 4. Half hydrogen gas and half nitrous gas. 5. Half azotic gas and half nitrous gas. 6. Half azotic gas and half oxygen gas. 7. Half azotic gas and half hydrogen gas. Pieces of flesh being introduced and the vessels well shut, a considerable redness appeared on the flesh in 1, 4, and 5. Second day, the flesh in 1 and 5 was particularly red; in the same manner it was in 2, 3, and 7, but in 4 and 6 it was yellow, reddish, and putrid. Third day, in 1 the flesh still remained of a beautiful red; less in 5; in 2 and 3 it was dark red; in 4 brownish and putrid, somewhat liquid; in 7 reddish, but not putrid. Fourth day, the pieces were all dark brown or dark red; in 1 and 5 however, it had the best appearance. Two months after the flesh appeared dark yellow brown, except in 1 and 5, in which it was dark red. In this state it remained unaltered during fourteen months.

Exp. 6. A small bottle, A, being filled above quicksilver with ammoniacal gas, a piece of beef was introduced, that had been well dried before with blotting paper; but notwithstanding the moisture of the flesh having absorbed some gas, it became necessary to admit a new portion. Having shut the opening with a stopple covered with a wet piece of bladder, I observed the flesh to have remained unaltered some hours after. Another bottle, B, was likewise filled over quicksilver with muriatic gas, and a piece of beef being introduced in the same manner as before, it became whitish and moist. A bottle, C, was filled with oxydated muriatic gas, and the flesh brought into it, acquired soon after a white colour, while the gas was partly deprived of its yellow colour, and a liquor was perceived, which was probably common muriatic acid. Two bottles signed signed D and E were filled in the same manner with hepatic gas, into which were immitted pieces of beef, which soon became blackish. At last I filled a bottle, G, with phosphoric gas, in which the flesh retained its colour; but in the bottle H, which I had filled with three parts of phosphoric gas and one part of hepatic gas, the flesh became grey some time after. On the second day, I found the flesh in D, E, and F, had become blackish, whereas it had remained unaltered in the rest. Third day, the flesh in G and H was pretty fresh, but in A it was particularly blackish, in C white, and in D and E yellowish, in B evidently putrid. Sixth day, in G and H the flesh appeared dry and darker, as before, though it was still red. In A and B it was grey and blackish, in C yellowish, in D and E dark grey. The flesh being not much altered the following day, I took it out on the ninth day, and made the following observations. In A it was of a glossy black, dry and solid, its smell a little ammoniacal; three-fourths of the gas were absorbed by water, the remaining gas extinguished a candle, and phosphorus did not shine in it. In B the flesh had not a very putrid smell, but it was soft and brown; the gas extinguished a candle, but phosphorus emitted fumes in it. In C the flesh was yellowish and soft. In D the flesh had an hepatic smell and was blackish; the gas had lost nothing of its properties, and proved inflammable. In E the flesh was black, solid, and had the hepatic smell; the gas was no more inflammable, and phosphorus emitted fumes in it. On opening all these bottles under water, a part of them were filled with water, so that some part of the gas seemed to have been absorbed. But on opening the bottle G, no water had got in; the flesh had a caseous smell and was of a dark red colour, the gas was inflammable without causing an explosion. The same appearances were observed in the bottle H.

Exp. 7. I prepared very pure oxydated muriatic gas and intercepted it in bottles, each containing 15 cubic inches, part of which were already filled with other species of gasses, and thus I obtained the following mixtures: No. 1. Half oxydated muriatic gas and half azotic gas. 2. One-fourth oxydated muriatic gas and three-fourths hydrogen gas. 3. One-twentieth oxydated muriatic gas, one-fourth azotic gas, and about three-fourths hydrogen gas. 4. Half oxydated muriatic gas and half oxygen gas. 5. Oxydated muriatic gas. 6. Half oxydated muriatic gas and half hydrogen gas. Having introduced into them pieces of beef, I shut the vessels as closely as possible,

In

In 5 the flesh had already grown white seven or eight minutes after, and also the flesh in 1, 4, and 6 was gradually discoloured. On the second day, all the flesh except in 3 was perfectly white; but towards evening, having been exposed to the action of the sun, it had changed to a dark yellow colour; in 3 however it was yellowish white. Third day, the flesh became brownish; in 3 it was yellowish red. The following days it received a darker colour. The temperature was from 12 to 22 degrees. Ten days after, on examining the whole, I made the following remarks:

No. 1. About one-third its volume of water penetrated into the bottle; the flesh was solid but friable, and had a particular though not putrid smell; the remaining gas troubled the lime-water, and extinguished a candle.—No. 2. The flesh was about the same as in No. 1; the gas made lime-water turbid, and was inflammable.—No. 3. The flesh was solid, hardly friable, and nearly of the same smell as No. 1; the gas was inflammable.—No. 4. On opening the vessel a quantity of water entered it; the flesh was found as in No. 1; lime-water absorbed part of the gas and became turbid; a candle burned in it.—No. 5. The flesh was easily friable, a little greasy, had a particular but not putrid smell; lime-water became turbid by the remaining gas, which also extinguished a candle.—No. 6. The flesh was nearly as in No. 1; the gas troubled lime-water, and it was inflammable, causing at the same time a slight explosion.

Exp. 8. The following bottles were filled with gas, into which pieces of beef were introduced as before.—A, nitrous gas; B, carbonic gas; C, half nitrous gas and half carbonic gas; D, hydrogen gas; E, azotic gas; F, atmospherical air; G, oxygen gas. Several hours after I found the flesh in A, B, F, and G, redder than that in D and E. Second day, towards the evening, it was beautifully red in A and C; less red in B, D, and E; in F it was dark red, and somewhat putrid; in G however blackish, greenish, and in all appearance more putrid than that in F. Third day, in the bottles A, B, C, D, and E, the flesh had grown darker and dry; in F it looked putrid and of a bluish dark brown colour; however, it appeared more putrid in G. The following days the flesh became darker and the putrefaction seemed to have increased in F and G. The temperature was between 14—20° Reaum. Two months after the flesh in A became yet darker without putrefaction; it was less red in B and C; in D and E the colour was brown; in F and G it was very putrid, yellowish, brown, and black.

A year after, I found the flesh in A and C to be the best preserved; whereas, in the other vessels, it was more or less yellow, brown, and blackish. Fifteen months after I opened the vessels under water, and observed as follows: A. Much water entered the bottle, the flesh was solid, of a dark red colour, and had a slight caseous smell.—B. The flesh had a caseous smell, was soft and greasy; the gas extinguished candle-light, and troubled lime-water.—C. The flesh was putrid, grey, green, reddish, but solid; it extinguished a candle immediately. D. The flesh had a slightly putrid smell, was friable, the gas was not inflammable. E. The smell of the flesh was somewhat putrid, but it was solid; the gas extinguished light, but caused little turbulency in lime-water. F. The flesh was greasy, of a very nauseous putrid smell, which it had likewise communicated to the gas. G. The flesh was very soft and greasy, it had a very disagreeable smell; lime-water became very turbid by the admixture of the remaining gas. The quantity of the gas was found to be more or less diminished in the bottles, as appeared from the water entering into the vacuum. All the remaining gasses were tried by the eudiometer.

Exp. 9. Several glass bells, provided at the upper extremity with small necks and openings, with stopples well luted, on which pieces of flesh had been previously applied, were filled with different gasses; as, No. 1. with hydrogen gas. 2. Nitrous gas. 3. Azotic gas. 4. Atmospherical air. 5. Oxygen gas. The pieces of flesh in 2 and 3 acquired immediately a beautiful red colour, which continued in 2 the following day; but in 5, and also in 4, it had a darker colour, and a putrid smell was to be perceived. Third day, in 2 the flesh was still red, and of a fresh appearance; in 3 darker, but without a putrid smell; in 4 it was dark brown, and of a putrid smell; and in 5 the water had entered into the bell, surrounding part of the flesh, where it was whitish, but in the gas itself it was dark red, of a putrid appearance. I filled the bell again with oxygen gas. Fifth day, no particular alteration was observed; however, of the oxygen gas about one-quarter was absorbed; and likewise the nitrous gas was diminished, so that the water touched the flesh. A month after this I found the flesh in 1 yellow red, but the water surrounding the bell had entered so far that the piece of flesh was quite immersed in it. In the same manner I found it in 2, but the flesh was hard, and of a whitish colour. In 3, less water had entered, and the flesh was still red but moist.

In

In 4, only part of the flesh was covered with water; it was dark or blackish, and liquefied. In 5, the flesh was for the most part liquefied, and the water nearly filled up the bell, on which account I had, from time to time, admitted a new portion of oxygen gas. In the seventh week, the water in 1 had risen so much that only one-seventh of the bell remained vacant; the flesh, which was under water, seemed however to be solid, but on being touched I found it to be soft and greasy; it was of a white colour, and of a putrid smell. In 2, the flesh appeared solid, white, and had no particular smell. In 3 the gas had a putrid smell like the flesh, which was white and soft. In 4, the water filled three-fourths of the bell, the flesh was blackish and very putrid. In 5, the flesh was nearly liquefied, the remaining gas had a very fetid smell; the eudiometer showed, in all the gases, more or less diminution, &c.

Exp. 10. Fresh pieces of beef were put into several glass bottles, containing the following gasses: No. 1. Nitrous gas. 2. Carbonic gas. 3. Hydrogen gas. 4. Azotic gas. 5. Atmospherical air. 6. Oxygen gas. Second day the redness of the flesh in 1 had much increased, and likewise in 2, 5, and 3; but in 6 the colour was less vivid, it appeared a little yellowish. Third day, except in 6, where the flesh began to dry up, no alteration was observable. Fourth day, in 6 the flesh looked extremely putrid; in 1 it was fresh and red; less in 2, 3, and 4; in 5 it appeared yellowish, with increasing putrefaction. Eighth day, in 1 the flesh was not yet altered; and in 2, 3, and 4, no certain sign of putrefaction could be perceived; in 5 it was putrid; in 6 the putrefaction had increased, though it still remained solid. Twelfth day, in 1 the flesh continued to be red; in 2, 3, and 4, it had a light red colour; in 5 it began to liquefy, and was of a bluish colour; in 6 it was very putrid, blackish, and liquid. A month after putrefaction was perceptible in all the pieces except in 1, which I still found fresh after a space of four months; it was solid, and had only a very slightly caseous smell. In 2, 3, and 4, the colour was more or less blackish, the smell putrid; but in 5, the flesh was soft, moist, and liquid, and emitted a putrid stench; all which circumstances were still more obvious in 6. The vessels had, during the experiment, always remained in the shade; the middle temperature was, in the first weeks, from 11 to 16° Reaum.; but afterwards it was only from 10 to 13°. I exposed the putrid pieces of flesh to oxydated muriatic gas, by which white fumes were raised; the putrid smell disappeared, instead of of which a caseous smell remained which could be no farther destroyed.

Exp. 11. I took pieces of flesh, which had been already kept twelve days in a cellar, and were almost putrid, and introduced them into glass bells, which I had filled with the following gasses: A. Nitrous gas. B. Azotic gas. C. Hydrogen gas. D. Half atmospherical air and half oxygen gas. The flesh in A. became considerably redder, but the other pieces did not change their colour. Second day, I found the flesh in A. of a beautiful red; in B. and C. it was not much changed; in D. however, it was of a dark colour, and putrid. The flesh in A. retained its colour for a considerable time after; but in B. and C. and still more in D. the flesh continued to putrify. The bells were standing in the shade, at a temperature of 10 to 15° Reaum.

On comparing the experiments described here, I think myself enabled to draw the following results.

1. *Oxygen gas* surpasses all other gases in promoting putrefaction within the shortest time, and rendering it most perfect; for notwithstanding that the flesh assumes a redder colour soon after its being immersed in it, the marks of putrefaction become visible within twenty-four hours, the flesh growing yellow or brown, with small black spots on its surface, and bluish black and liquid. Part of the oxygen is hereby decomposed, and carbonic gas is produced. There is very little or no hydrogen gas generated during the process, a circumstance which seems to render the opinion of Priestley very doubtful, as is likewise intimated by Berthollet, who says, that the gas produced by putrefaction contains much carbon but no hydrogen.

2. *Azotic gas* neither particularly promotes nor hinders putrefaction. Flesh exposed to it retains at first its natural colour; but eight, sixteen, or more days after, the external marks of putrefaction gradually appear, which, slowly advancing, never becomes perfect; the flesh remaining more or less solid, grows greasy, with a caseous putrid smell, which however is by no means so strong as in oxygen gas, and only a small quantity of carbonic gas is produced.

3. *Hydrogen gas* has nearly the same effect on flesh as azotic gas; and if in some experiments the flesh appears better preserved in it, we should rather ascribe this circumstance to the nitrous fumes with which it is sometimes a little impregnated; it is, however, in some measure deprived of its inflammability, and carbonic gas is evolved. From my experiments it appears, that Signor Brugnatelli's

 opinion opinion is by no means confirmed, who thinks this gas to be particularly antiseptic. It likewise results from my experiments, that the accession of air and water is not absolutely necessary for producing putrefaction, as is commonly believed.

4. *Carbonic gas* but slightly reddens flesh immersed in it; and though it prevents in some measure the progress of putrefaction, yet within four or five weeks the flesh arrives at the same degree of corruption as in azotic and hydrogen gas; it becomes soft, greasy, and if not dried up it receives a particular caseous and putrid smell.

5. *Hepatic gas* imparts a yellow, grey, and blackish colour to the flesh, and in its other effects it resembles hydrogen gas.

6. *Phosphoric gas* seems to be somewhat more antiseptic than common hydrogen gas, but on the whole its effects on flesh are nearly the same.

7. The effects of *ammoniacal gas* and of *muriatic gas* on flesh are not easily to be determined, as they are in some measure deprived of their gasiform state, combining themselves with the moisture of the flesh, and thus forming liquid ammonia and muriatic acid, both which act in a different manner from the gas.

8. The *oxydated muriatic gas* is partly changed into the acid of the same name by the moisture of the flesh; and in this state, and also as gas, it discolours flesh, which sometime after becomes soft. Its white colour, however, is changed into yellow by the action of sun-light.

9. *Nitrous gas* is above all others possessed of the most eminent antiseptic power; and flesh grows in it beautifully red, which colour it retains for several weeks; it then passes over into dark red, and keeps its fresh appearance for above a twelvemonth. It never becomes perfectly putrid, but only obtains at last a slight caseous smell. Flesh already attacked by putrefaction is preserved for a long time in this gas, which however in this manner, by degrees, loses its property of yielding red nitrous fumes, when mixed with oxygen gas.

10. *Atmosphcrical air* is to be considered as a mixture of oxygen gas and azotic gas, on which account the progress of putrefaction goes on more slowly than in pure oxygen gas; nor does it arrive at such a degree as in this gas.

11. In mixtures consisting of azotic gas, hydrogen gas, and carbonic gas, with a portion of nitrous gas, the last evidently exerts its antiseptic quality on the flesh contained in them.

12. Mix-

12. Mixtures of other gasses with hydrogen gas and azotic gas, prove not particularly antiseptic.

13. Mixtures of azotic gas and hydrogen gas, or of oxygen gas or atmospherical air, with carbonic acid, have no considerable and lasting antiseptic quality.

14. Oxydated muriatic gas mixed with different other gasses, has antiseptic effects, but is easily changed into acid by the moisture of the flesh.

